# Multi-technique comparison of atherogenic and MCD NASH models highlights changes in sphingolipid metabolism

**DOI:** 10.1038/s41598-019-53346-4

**Published:** 2019-11-14

**Authors:** Sophie A. Montandon, Emmanuel Somm, Ursula Loizides-Mangold, Claudio de Vito, Charna Dibner, François R. Jornayvaz

**Affiliations:** 1 Service of Endocrinology, Diabetes, Hypertension and Nutrition, Department of Internal Medicine, Geneva University Hospitals/University of Geneva, Geneva, Switzerland; 2 Department of Cell Physiology and Metabolism, University of Geneva, Geneva, Switzerland; 3 Diabetes Center, Faculty of Medicine, University of Geneva, Geneva, Switzerland; 4 Institute of Genetics and Genomics in Geneva (iGE3), University of Geneva, Geneva, Switzerland; 5 Division of Clinical Pathology, Geneva University Hospitals, Geneva, Switzerland

**Keywords:** Mass spectrometry, Metabolic syndrome

## Abstract

Lipotoxicity is a key player in the pathogenesis of nonalcoholic steatohepatitis (NASH), a progressive subtype of nonalcoholic fatty liver disease (NAFLD). In the present study, we combine histological, transcriptional and lipidomic approaches to dissociate common and specific alterations induced by two classical dietary NASH models (atherogenic (ATH) and methionine/choline deficient (MCD) diet) in C57BL/6J male mice. Despite a similar degree of steatosis, MCD-fed mice showed more pronounced liver damage and a worsened pro-inflammatory and pro-fibrogenic environment than ATH-fed mice. Regarding lipid metabolism, the ATH diet triggered hepatic counter regulatory mechanisms, while the MCD diet worsened liver lipid accumulation by a concomitant increase in lipid import and reduction in lipid export. Liver lipidomics revealed sphingolipid enrichment in both NASH models that was accompanied by an upregulation of the ceramide biosynthesis pathway and a significant rise in dihydroceramide levels. In contrast, the phospholipid composition was not substantially altered by the ATH diet, whereas the livers of MCD-fed mice presented a reduced phosphatidylcholine to phosphatidylethanolamine (PC/PE) ratio and a strong depletion in phospholipids containing the sum of 34–36 carbons in their fatty acid chains. Therefore, the assessment of liver damage at the histological and transcriptional level combined with a lipidomic analysis reveals sphingolipids as shared mediators in liver lipotoxicity and pathogenesis of NASH.

## Introduction

Nonalcoholic fatty liver disease (NAFLD) is a major cause of chronic liver disease, affecting approximatively 25% of the general population^1^. Nonalcoholic steatohepatitis (NASH) is a progressive subtype of NAFLD, which can result in cirrhosis and even hepatocellular carcinoma, thus representing an initial step in liver-related mortality^2,3^. NASH is histologically characterized by the presence of hepatic steatosis, hepatocyte ballooning associated with inflammation, with or without fibrosis^4^. Yet, fibrosis has been shown to be the most important histological predictor of disease-related mortality^5–7^. It is now well accepted that the NAFLD/NASH pathogenesis is due to “multiple hits” derived from the gut and the adipose tissue, leading to hepatic inflammation and lipid accumulation^8^. In turn, lipotoxicity causes mitochondrial dysfunction, oxidative and endoplasmic reticulum (ER) stress, sustained inflammation, hepatic stellate cell activation and eventually increases fibrous matrix deposition and hepatocyte death^9,10^.

NAFLD is often associated with obesity, type 2 diabetes, insulin resistance, hyperlipidemia and hypertension^1^ and therefore is sometimes considered as the liver manifestation of the metabolic syndrome. Nevertheless, genetic predisposition, fructose- and cholesterol-rich diets, visceral adiposity or dyslipidemia can favor the onset of “lean NAFLD” in non-obese patients^11,12^. Continuous monitoring of this multifactorial disease is challenging in humans, because of the invasiveness of liver biopsies and the slow rate of progression. The currently rising prevalence of NAFLD and the complexity of the disease have spurred the development of surrogate animal models^13–15^. Ideally, the model should rapidly reproduce the pathological features of human NASH and progress to more advanced stages of the disease such as hepatocellular carcinoma. Although various genetic and dietary rodent models have been developed, recapitulating the heterogeneity of the human disease requires the combined use of multiple models^16^.

In the present study, we combine multiple approaches to dissociate common and specific alterations caused by the classic cholesterol/cholate-rich atherogenic (ATH) diet and the methionine and choline deficient (MCD) diet. Both models induce lipid accumulation in the liver, but the nature of the accumulating metabolite is different. While both diets lead to an increase in hepatic free fatty acids^17,18^, the livers of MCD-fed mice are enriched in triglycerides^17,19–21^ whereas the livers of ATH-fed rodents predominantly accumulate cholesterol^18,22^. Due to increased energy expenditure^20,23^, ATH- and MCD-fed mice do not gain body weight and do not develop global insulin resistance^18,24^. Yet, they present hepatic insulin resistance^18,25,26^ and are appropriate to study NASH pathogenesis in the absence of confounding parameters (systemic insulin resistance or obesity). By combining classical histology, pathological evaluation and gene expression analysis, we show that both dietary models rapidly recapitulate the liver manifestation of human NASH. In addition, our lipidomic analysis strongly highlights the hepatic accumulation of sphingolipids, including ceramides and their derivatives, as a lipotoxic signature in both models.

## Results

### Blood, adipose tissues and gut parameters of ATH- and MCD-induced NASH models

We challenged C57BL/6J male mice with two different diets: a diet deficient in methionine and choline (MCD) for 7 weeks or an atherogenic (ATH) diet rich in lipids, cholesterol and cholate for 12 weeks. From a global point of view, the MCD diet drastically reduced mice body weight, white adipose tissue depot mass and adipocyte size (Table 1, Supplementary Fig. S1A). In contrast, brown adipose tissue (BAT) mass was only modestly diminished and even increased when expressed relative to body weight (Table 1). Mice fed the MCD diet had higher circulating levels of free fatty acids (FFA), but reduced plasma levels of glucose, insulin, total cholesterol and triglycerides compared to controls (Table 1). The ATH diet did not alter body weight, but moderately enlarged epididymal and inguinal white fat depots, while BAT mass decreased (Table 1). Histologically, BAT from both ATH- and MCD-fed mice contained fewer and smaller lipid droplets with intense cytoplasmic staining (Supplementary Fig. S1B). Mice fed an ATH diet presented no change in glycaemia and FFA plasma levels, but a decrease in insulinemia, HOMA-IR (Homeostatic Model Assessment for Insulin Resistance^27^) index and triglyceridemia concomitant with a rise in circulating total cholesterol levels (Table 1). Both diets substantially altered the gene expression of ileal nutrient transporters. The lipid-rich ATH diet increased *Cd36* and *Fabp6* mRNA expression, while the carbohydrate-rich MCD diet upregulated mRNAs for glucose (*Slc5a1*) and fructose (*Slc2a5*) transporters (Supplementary Fig. S2A). In the MCD group, cholesterol (*Npc1l1*) and peptide (*Slc15a1*) transporter levels were also increased, whereas lipid transporters were slightly downregulated (Supplementary Fig. S2A). Furthermore, the MCD diet highly affected ileal morphology by reducing both villus and crypt sizes compared to the CTRL and ATH groups (Supplementary Fig. S2B–D).

**Table 1:** Body weight, adipose tissues mass and blood parameters.

	CTRL		ATH		MCD	
Initial body weight [g]	24.18	1.45	24.03	1.74	24.33	2.01
Final body weight [g]	27.55	1.63	26.91	1.56	16.35	0.95^*,#^
Total cholesterol [mmol/L]	1.70	0.12	2.96	0.50*	0.52	0.19^*,#^
Triglycerides [mmol/L]	0.71	0.13	0.52	0.13*	0.48	0.10*
Free fatty acids [mmol/L]	0.63	0.14	0.59	0.09	1.00	0.41^*,#^
Insulin [ng/ml]	0.38	0.09	0.31	0.08*	0.07	0.02^*,#^
Glucose [mmol/L]	9.95	1.02	9.05	2.01	4.60	1.71^*,#^
HOMA-IR index	28.96	6.37	21.76	9.24*	2.94	1.70^*,#^
eWAT [%BW]	1.40	0.23	1.80	0.50*	0.14	0.09^*,#^
eWAT adipocyte size [µm2]	1791.55	327.38	1748.77	345.04	286.01	62.42^*,#^
iWAT [%BW]	0.67	0.16	0.84	0.20*	0.26	0.08^*,#^
BAT [mg]	72.02	10.18	57.39	6.46*	62.38	7.86*
BAT [%BW]	0.26	0.03	0.21	0.02*	0.37	0.05^*,#^

Metabolic parameters CTRL, ATH and MCD groups (mean in bold and standard deviation). One-way ANOVA: *p value ATH/MCD *vs* CTRL < 0.05, #p value MCD *vs* ATH < 0.05.

### Evaluation of the liver damage induced by ATH and MCD diets

**Figure 1:**
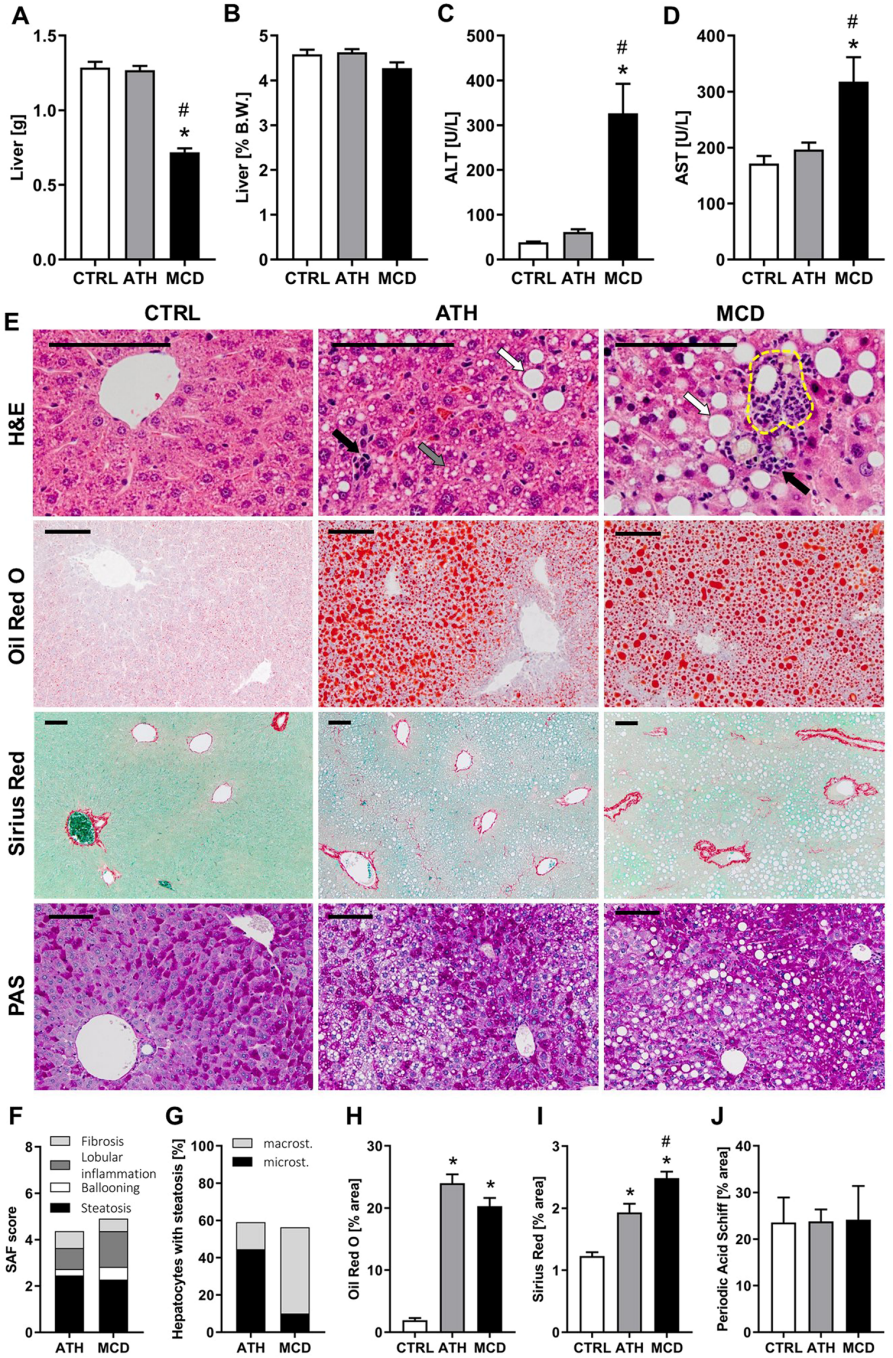
ATH and MCD diets induce similar degree of steatosis, but different NASH severity. Liver weight expressed in grams (**A**) and as percentage of body weight. (**B**) Plasma levels of liver enzymes: ALT (**C**) and AST. (**D**) Liver histology: hematoxylin and eosin (H&E), Oil Red O, Sirius Red and periodic acid Schiff (PAS). (**E**) SAF score with Steatosis, Activity (lobular inflammation + ballooning) and Fibrosis. (**F**) Evaluation of the percent of hepatocyte containing micro- and macrosteatosis. (**G**) Quantification of lipids (**H**), collagen (**I**) and glycogen (**J**) expressed as percent of area stained respectively with Oil red O, Sirius Red and PAS. Scale bars 100 µm, black arrows: immune cell infiltration, white arrows: macrosteatosis, grey arrow: microsteatosis, yellow dashed circle: lipogranuloma. One-way ANOVA: *p value ATH/MCD *vs* CTRL < 0.05, ^#^p value MCD *vs* ATH < 0.05.

Liver weight was unchanged in ATH-fed mice and significantly reduced in MCD-fed mice (Fig. 1A), yet no change was observed relative to body weight (Fig. 1B). Circulating liver enzymes ALT and AST were strongly elevated in MCD-fed mice, but stayed unaltered in ATH-fed mice when compared to CTRL counterparts (Fig. 1C,D). Nevertheless, pathological evaluation of liver sections, stained with hematoxylin and eosin (H&E), revealed that both diets induce hepatocyte ballooning and lobular inflammation (Fig. 1E,F). In addition, lipogranuloma (*i*.*e*. inflammatory nodules composed of macrophages surrounding a lipid droplet) were observed in MCD-fed mice livers (yellow dashed circle in Fig. 1E). Quantitatively, almost 60% of hepatocytes accumulated lipids in both groups (Fig. 1G) and more than 20% of the liver surface area was taken over by lipids (Fig. 1E ‘Oil red O’ staining and Fig. 1H). Despite similar degrees of steatosis in the ATH and MCD groups, livers of ATH-fed mice mostly presented numerous small lipid vesicles (*i*.*e*. microsteatosis, Fig. 1G, grey arrow in Fig. 1E), while livers of MCD-fed mice had predominantly a single large lipid droplet (*i*.*e*. macrosteatosis, Fig. 1G, white arrows in Fig. 1E). In both NASH models, collagen deposition was significantly increased compared to controls (Fig. 1E ‘Sirius Red’ and Fig. 1I), but the MCD group showed a more pronounced accumulation than the ATH group (Fig. 1I). Finally, periodic acid Schiff (PAS) staining illustrated no major change in glycogen storage in both NASH models compared to CTRL mice (Fig. 1E,J).

Taken together, these results confirm that both diets induced the liver manifestations of NASH, *i*.*e*. steatosis, hepatocyte ballooning, lobular inflammation as well as fibrosis. Despite similar degrees of lipid accumulation between ATH- and MCD-fed mice, liver damage was more pronounced in the MCD group, accompanied by tissue fibrosis and elevated liver enzymes.

### Transcriptional changes in hepatic enzymes and transporters behind the lipid accumulation and pro-fibrogenic environment of ATH- and MCD-fed mice

**Figure 2:**
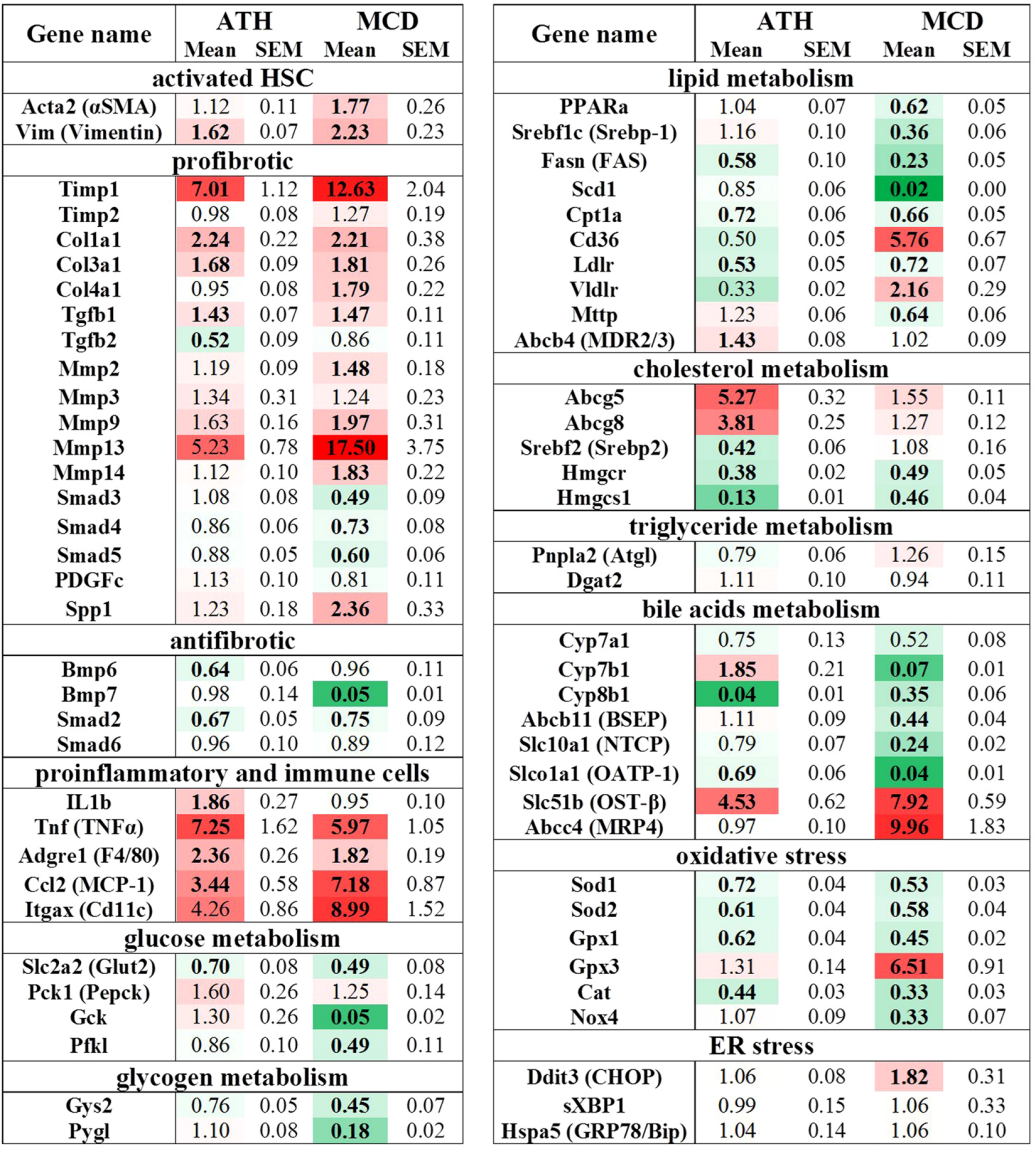
Real-time quantitative PCR of hepatic lipid genes of ATH- and MCD-fed mice. Hepatic relative gene expression (mean and SEM) ordered by metabolic function. Normalized to *RPS29*. Mean values in bold: p value *vs* CTRL group < 0.05. Color code according to the LOG2 of the mean from dark green (highly downregulated *vs* CTRL group), white (no change) to dark red (highly upregulated).

To elucidate the molecular pathways underlying the manifestations of NASH in ATH- and MCD-fed mice, we studied hepatic transcriptional changes through quantitative PCR with a special focus on genes involved in fibrogenesis, inflammation and lipid homeostasis (Fig. 2, upregulated genes in red, downregulated genes in green). Both, ATH and MCD groups presented an upregulation of *Vim* and *Acta2* mRNA, classical markers of activated myoblast-like hepatic stellate cells (HSCs). In addition, pro-fibrogenic markers (*Timp1*, *Col1a1*, *Col3a1*, *Tgfb1*, *Mmp13*) were overexpressed in the livers of ATH- and MCD-fed mice. In line with the histological fibrosis quantification (Fig. 1I), a subset of pro-fibrogenic markers (*Timp1*, *Spp1*, *Col4a1* and *Mmp2*,*3*,*13* and 14) were further increased in MCD-fed mice compared to ATH-fed mice. Moreover, the overexpression of pro-inflammatory mediators (*Tnf*, *Ccl2*) and macrophage markers (*Adgre1*, *Itgax*) confirmed the lobular inflammation histologically observed. Interestingly, the expression of anti-fibrogenic markers of the Bone Morphogenic Protein (BMP) family Bmp6 and Bmp7 are differently regulated in the MCD and ATH groups. While *Bmp7* is specifically and drastically abrogated in MCD-fed mice, *Bmp6* is significantly downregulated in ATH-fed mice. As a common signal transducer of TGF and BMP, SMAD2, previously reported as anti-fibrotic^28,29^, is significantly downregulated in both ATH- and MCD-fed mice.

Transcriptional analyses reflected that the MCD diet led to severe hepatic lipid accumulation due to impairment in lipid export (decreased *Mttp* expression), associated with increased import of lipids (*Cd36*, *Vldlr*), originating from lipolysis in the WAT, as highlighted by WAT shrinkage and rise in circulating FFAs (Table 1). This imbalance in hepatic lipid transport was accompanied by a decrease in *de novo* lipogenesis (*Srebf1c*, *Fasn*, *Scd1*) and fatty acid oxidation (*PPARa*, *Cpt1a*), with no major change observed in enzymes gating triglyceride turn-over (*Pnpla2*, *Dgat2*). The ATH group also showed a reduction in *de novo* fatty acid synthesis (*Fasn*) and oxidation (*Cpt1a*), but in contrast to the MCD group, the expression levels of fatty acid translocase CD36 (*Cd36*) and lipoprotein transporters (*Ldlr*, *Vldlr*) were downregulated. Moreover, the ABC transporter MDR3 (*Abcb4*), responsible for maintaining an appropriate phospholipid concentration in the bile, was upregulated and lipid exporter (*Mttp*) was slightly increased in the ATH group.

Both ATH- and MCD-fed mice exhibited a downregulation of cholesterogenic genes (*Hmgcr*, *Hmgcs1*), but only ATH-fed mice presented a significant overexpression of the apical cholesterol transporters *Abcg5* and *Abcg8*. Concerning bile acid (BA) synthesis, *Cyp7a1* (the rate-limiting enzyme) showed a tendency to decrease in both models. *Cyp8b1* (driving the classic pathway of BA synthesis) was downregulated in ATH- and MCD-fed mice. In contrast, *Cyp7b1* (driving the alternative pathway of BA synthesis) was upregulated in the ATH group, but drastically reduced in the MCD group. With regard to BA transport, the apical exporter *Abcb11* was downregulated only in MCD mice. Basolateral importers (*Slc10a1* and *Slco1a1*) were downregulated in the ATH group and even more in MCD group while the basolateral exporter *Slc51b* was upregulated in both groups.

In term of glucose homeostasis, a decrease in *Glut2* expression was observed in both ATH- and MCD-fed mice. In addition, glucokinase (*Gck*), phosphofructokinase (*Pfkl*), glycogen synthase (*Gys2*) and glycogen phosphorylase (*Pygl*) were all specifically downregulated in the MCD group, in line with low glycaemia and insulinemia. In comparison, enzymes driving glucose and glycogen metabolisms were only poorly affected in the ATH-fed mice.

Almost all enzymes involved in oxidative stress showed a decreased expression in the livers of ATH- and MCD-fed mice (*Sod1*, *Sod2*, *Gpx1*, *Cat*), with a more pronounced downregulation observed in MCD compared to ATH group. In contrast, glutathione peroxidase 3 (*Gpx3*) and the ER stress marker *Chop* were specifically overexpressed in the MCD group.

To sum up, changes in gene expression indicate that in MCD-fed mice hepatic steatosis is cumulatively worsened by increased lipid uptake and reduced lipid export, this imbalance leading to a repression of *de novo* lipogenesis. In contrast, ATH livers present counter regulatory mechanisms against fatty acid and triglyceride accumulation (reduced import and synthesis, increased export) as well as against cholesterol accumulation (reduced synthesis and increased apical export). Nevertheless, these compensatory mechanisms are insufficient to counterbalance the continuous alimentary supply.

### Lipidomics of ATH- and MCD-fed mice reveals major changes in lipid homeostasis in the liver

**Figure 3:**
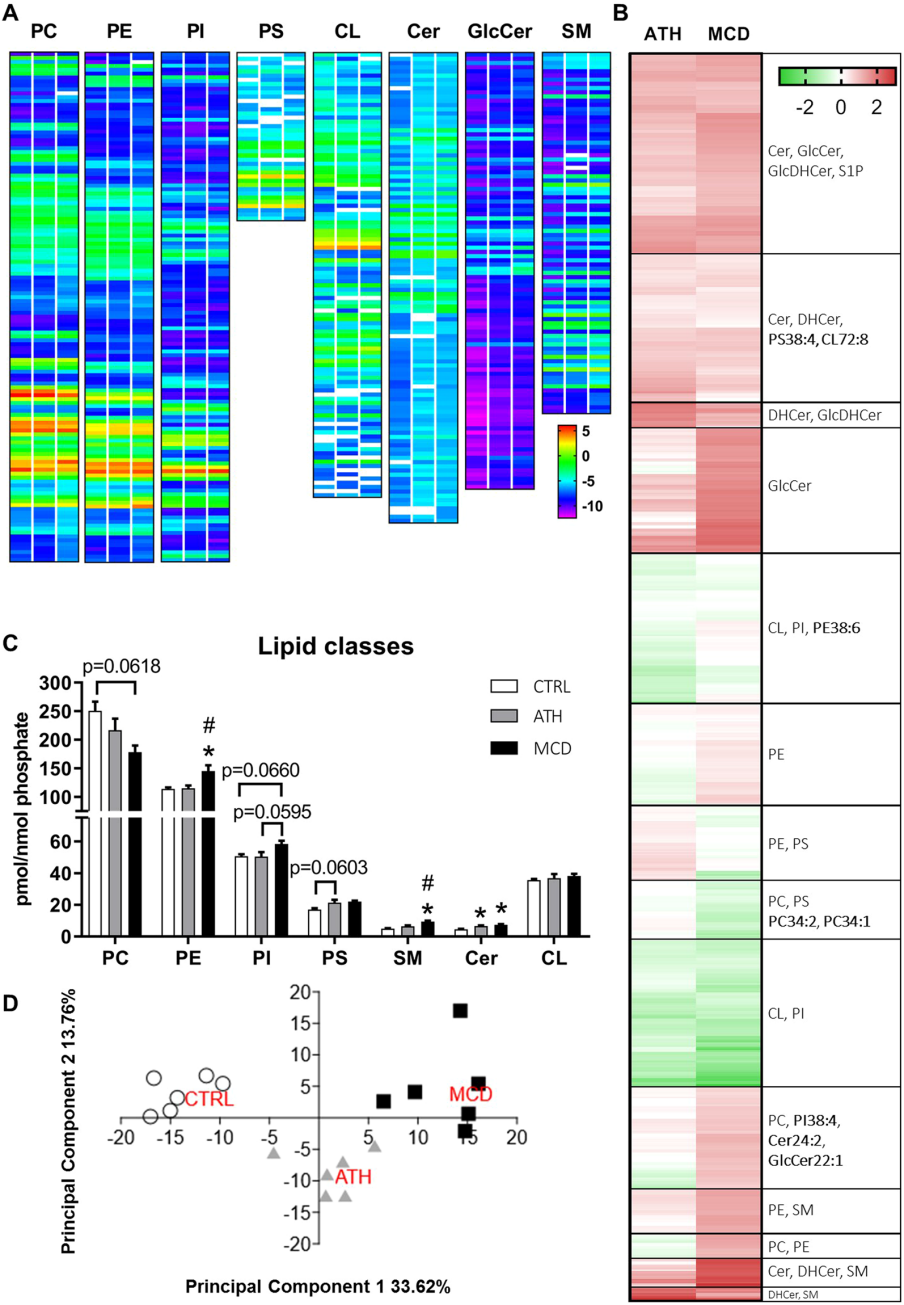
Lipidomic analysis of 944 lipid species of CTRL, ATH and MCD mouse livers. LOG2 of mean concentration of each lipid metabolite sorted by lipid class (PC, PE, PI, PS, CL, Cer, GlcCer, SM) and represented as heat maps. CTRL group = left column, ATH group = central column, MCD group = right column. (**A**) Hierarchical clustering of 801 lipid species presented as LOG2 of fold change *vs* Ctrl group and most represented lipid classes per cluster. (**B**) The predominant species per lipid group is shown in bold. Absolute concentration of lipid classes normalized to phosphate content. (**C**) Principal component analysis of the 801 lipid species with N = 6 animals per group. (**D**) PC: phosphatidylcholine, PE: phosphatidylethanolamine, PI: phosphatidylinositol, PS: phosphatidylserine, CL: cardiolipin, Cer: ceramide, GlcCer: glycosylceramide, SM: sphingomyelin, DHCer: dihydroceramide, GlcDHCer: dihydroglycosylceramide, S1P: sphingosine-1-phosphate, SL: sphingolipids. One-way ANOVA: *p value ATH/MCD *vs* CTRL < 0.05, ^#^p value MCD *vs* ATH < 0.05.

In order to evaluate qualitative changes in hepatic lipid composition, we analyzed a total of 944 lipid species by targeted mass spectrometry, among which 801 were detected in all groups (Fig. 3A). Hierarchical clustering analysis of the fold change *vs* the CTRL group (Fig. 3B) strongly highlighted the enrichment of sphingolipids (SL), whose overall content was significantly augmented in the ATH and MCD groups (Fig. 3C). Indeed, clusters regrouping dark red lipid species (*i*.*e*. more than 2 fold increase) mostly contain SL species. In contrast, lipid species underrepresented in both NASH models (in green in Fig. 3B) mostly belonged to the mitochondria-specific lipid class of cardiolipins (CL) or to the class of phosphatidylinositols (PI, Fig. 3B). The cluster of lipid species that were reduced in the MCD group (in green), but not differently regulated in the ATH group (in white, light green or light red) contained predominantly phosphatidylcholine (PC) phospholipids and in particular the most common species: PC34:2 and PC34:1 (Fig. 3B). Principal component analysis (PCA) of the lipidomic profiles showed clear separation between the three diets (Fig. 3D). Lipid classes grouped into Principal Component 1 mostly included sphingomyelin (SM), glycosylceramide (GlcCer) and dihydroglycosylceramide (GlcDHCer) and allowed to distinguish the three experimental conditions, with the ATH group being an intermediate between the CTLR and the MCD groups. On the other hand, Principal Component 2 was dominated by phospholipids (PL), especially PC-containing PL and ether-bond containing PL and separated ATH group from CTRL and MCD groups.

### Sphingolipid metabolism is dysregulated in both ATH- and MCD-NASH models

**Figure 4:**
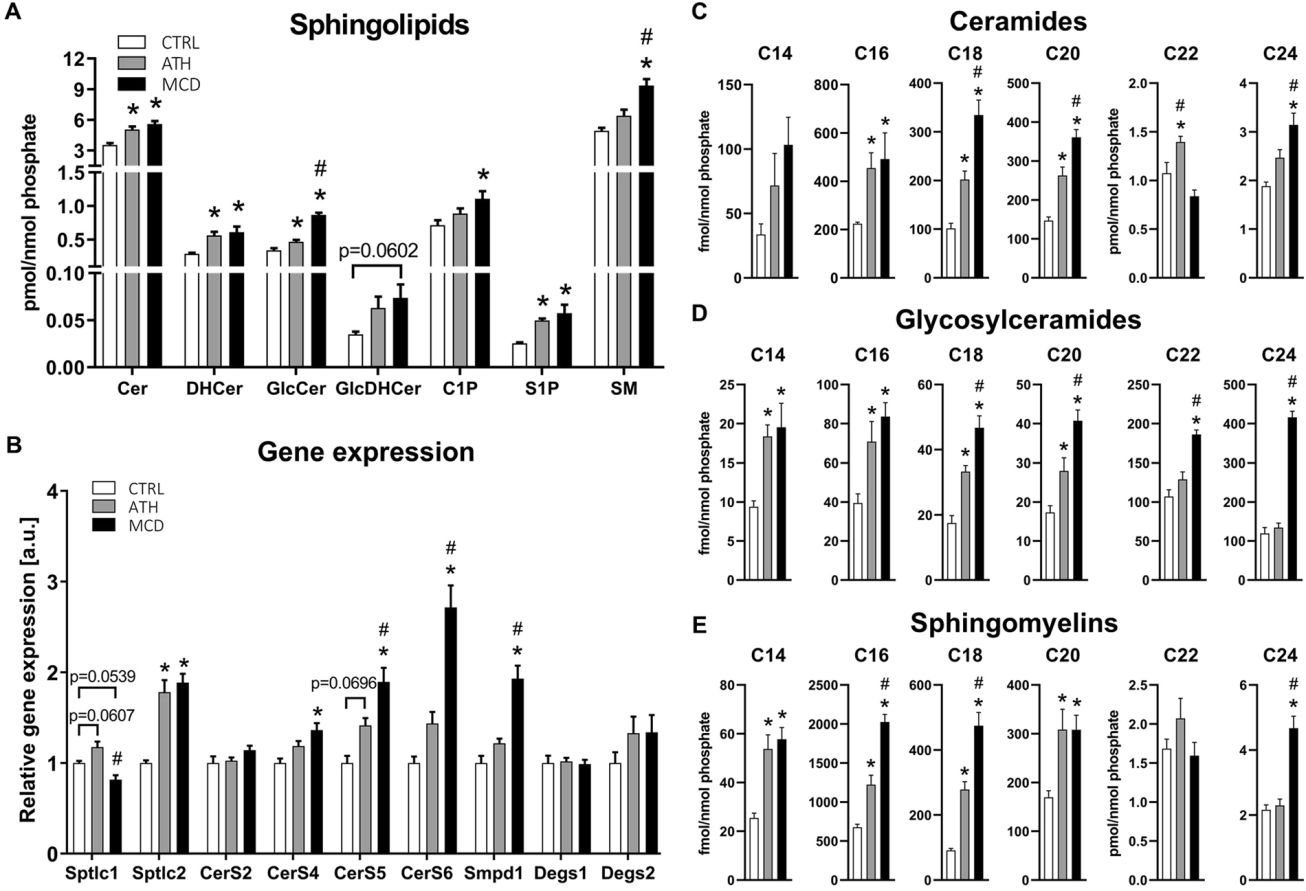
Sphingolipid metabolism is dysregulated in livers of ATH- and MCD-fed mice. Concentration of sphingolipid classes normalized to the phosphate content. (**A**) Gene expression of main enzymes involved in the ceramide biosynthesis pathways. (**B**) Concentrations of lipid species: ceramides (**C**), glycosylceramides (**D**) and sphingomyelins. (**E**) Cer: ceramide, DHCer: dihydroceramide, GlcCer: glycosylceramide, GlcDHCer: dihydroglycosylceramide, C1P: ceramide-1-phosphate, S1P: sphingosine-1-phosphate, SM: sphingomyelin. One-way ANOVA: *p value ATH/MCD *vs* CTRL < 0.05, ^#^p value MCD *vs* ATH < 0.05.

Ceramides (Cer) are central in the SL metabolism, since they can be glycosylated to become glycosylceramides or modified by a phosphocholine headgroup into sphingomyelins. Ceramides and glycosylceramides were both enriched in the ATH and MCD groups (Fig. 4A). This increase was observed for sphingolipid precursors (dihydroceramide (DHCer) and GlcDHCer) and mature forms (Cer and GlcCer). Importantly, the signaling sphingolipid sphingosine-1 phosphate (S1P) was significantly enriched in both NASH models (Fig. 4A).

To further understand the mechanisms involved in the accumulation of sphingolipids in both NASH models, we quantified the expression of genes involved in SL biosynthesis. *De novo* synthesis of ceramides is initiated by the serine-palmitoyl transferase (SPT), which has three subunits (*Sptlc1-3*). The *Sptlc2* subunit was significantly increased in both NASH models, whereas *Sptlc1* was only slightly upregulated in ATH-fed mice (Fig. 4B). Subsequently, ceramides are generated by a family of ceramide synthases (CerS) exhibiting different selectivity for fatty acyl CoAs according to chain length. Ceramide synthase 2 is the predominant CerS isoform in the liver and produces C20-C26 containing Cer^30,31^, among which Cer C24 is the most abundant Cer species (Fig. 4C). Even though *CerS2* was not upregulated in our NASH models (Fig. 4B), Cer C24 was significantly augmented in the MCD group and Cer C22 was elevated in ATH mice (Fig. 4C). Additionally, the glycosylated forms (GlcCer C22-C24) were significantly enriched in MCD-fed mice compared to the CTRL and ATH groups, with GlcCer C24 showing more than a 3-fold increase (Fig. 4D). The second most expressed hepatic CerS is *CerS4* that generates Cer C18-20. Cer C18-20 (Fig. 4C) and GlcCer C18-20 (Fig. 4D) were enriched in the livers of ATH- and MCD-fed mice, although only MCD-fed mice showed a significant increase in *CerS4* (Fig. 4B). The ubiquitously expressed enzymes *CerS5* and *CerS6* were significantly upregulated in the MCD group and were slightly augmented in the ATH group (Fig. 4B). These ceramide synthases produce Cer C14 (*CerS6*) and Cer C16 (*CerS5/6*) that were at least twice more abundant in the ATH and MCD groups than in the CTRL group (Fig. 4C,D). Note that DHCer desaturation by Degs1 and Degs2, which convert sphinganine-containing dihydroceramides into sphingosine-containing ceramides, does not seem to be altered by MCD and ATH diets (Fig. 4B). Ceramides are also generated by the rapid degradation of SMs by sphingomyelinases among which *Smpd1* was significantly upregulated in MCD livers (Fig. 4B). Surprisingly, even though SMs contain a choline head group all except SM C22 were significantly enriched in the livers of MCD mice after 7 weeks on a choline deficient diet compared to the CTRL group (Fig. 4E). Indeed, SM C16 and SM C18 respectively displayed a 3-fold and 5-fold increase compared to controls. In the ATH group, the overall SM content showed a mild increase (Fig. 4A). Albeit, C14-C20 SMs were significantly enriched with a respective 2-fold and 3-fold increase for SM C14 and SM C18 compared to the CTRL group (Fig. 4E).

To conclude, most sphingolipid species were enriched in the ATH and MCD NASH models. The accumulation of ceramides was more severe in the MCD group compared to the ATH group in line with the increased expression of ceramide synthases.

### Livers of MCD-fed mice are depleted in phosphatidylcholine and in phospholipid species with long, monounsaturated fatty acyl chains

**Figure 5:**
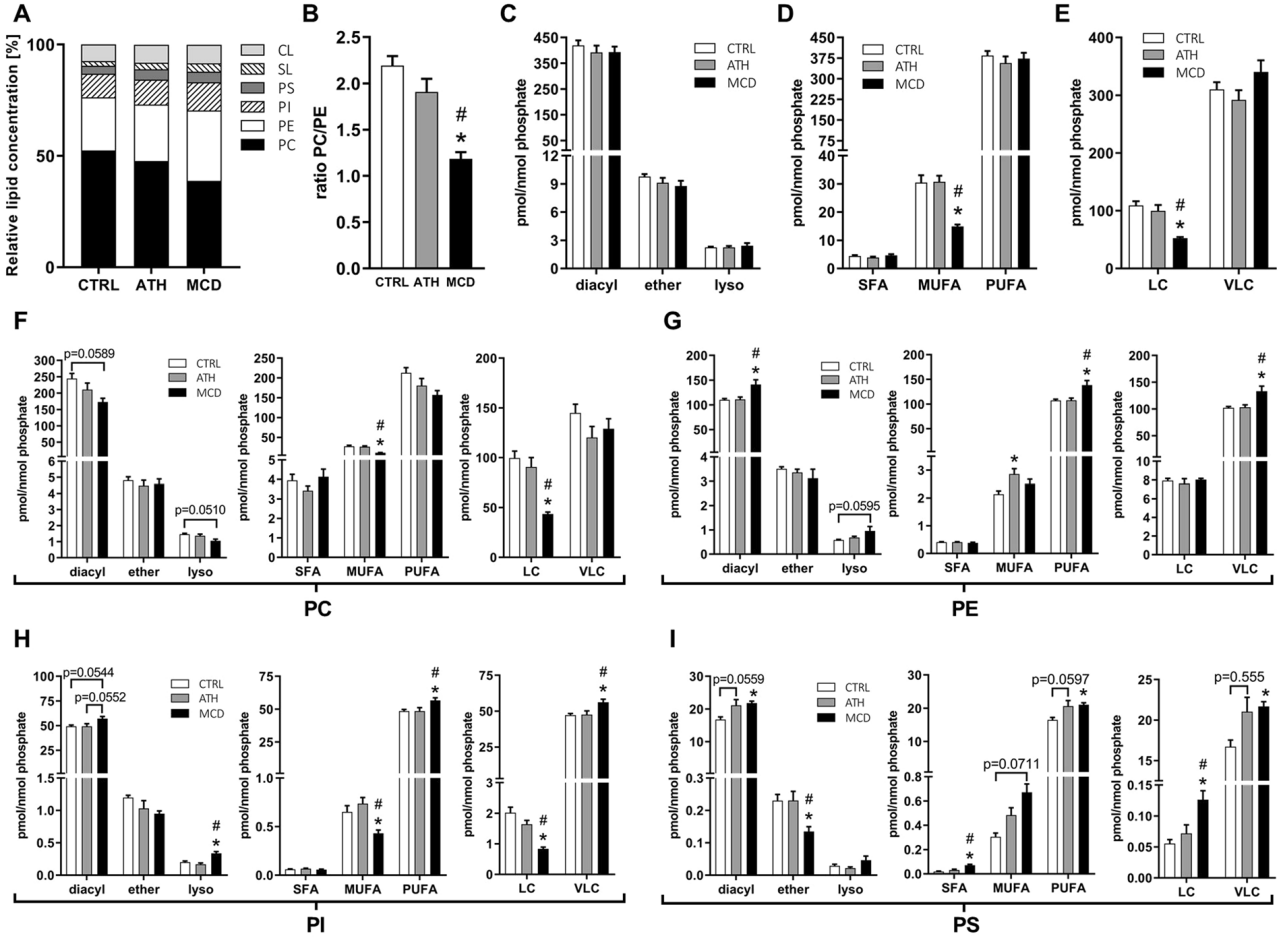
Liver lipidomics highlights effects of ATH and MCD diets on phospholipid homeostasis including phospholipid composition, fatty acid chain length and level of desaturation. Relative concentration of lipid classes. (**A**) Phosphatidylcholine to phosphatidylethanolamine ratio. (**B**) Total amount of PC, PE, PI and PS represented according to the nature of the fatty acid linkage (diacyl *vs* alkyl-acyl (ether) or monoacyl (lyso) (**C**), degree of saturation (**D**) or chain length (**E**). Same as in ‘C-E’ but separately for phosphatidylcholine (**F**), phosphatidylethanolamine (**G**), phosphatidylinositol (**H**) or phosphatidylserine. (**I**) PC: phosphatidylcholine, PE: phosphatidylethanolamine, PI: phosphatidylinositol, PS: phosphatidylserin1e, SL: sphingolipids, CL: cardiolipin, diacyl: diacylphospholipids, ether: alkyl-acyl-containing phospholipids, lyso: lysophospholipids, SFA: saturated fatty acids, MUFA: monounsaturated fatty acids (total sum of 1 double bond in both fatty acyl chains), PUFA: polyunsaturated fatty acids (total sum of 2–6 double bonds in both fatty acyl chains), LC: long chains (total sum of 28–34 carbons in both fatty acyl chains), VLC: very long chains (total sum of 36–44 carbons in both fatty acyl chains). One-way ANOVA: ^*^p value ATH/MCD *vs* CTRL < 0.05, ^#^p value MCD *vs* ATH < 0.05.

Phosphatidylcholine (PC) is the most abundant class of membrane phospholipids and in our analysis represented 52.44% of all lipids in control livers (Fig. 5A). Due to the choline deficiency of the MCD diet, PC levels were reduced (38.77% of all lipids) and membrane lipids became enriched in phosphatidylethanolamine (PE), phosphatidylinositol (PI) and phosphatidylserine (PS, Figs 3C and 5A). Therefore, the ratio between the two most predominant lipid classes PC/PE was significantly lower upon the MCD diet (Fig. 5B). The ATH diet showed a similar trend but this did not substantially influence the relative abundance of these two phospholipid classes (Figs 3C and 5A) and thus the PC/PE ratio (Fig. 5B). Diacyl phospholipids are the most common membrane lipid species (Fig. 5C) and their increase/decrease was representative of the overall total class abundance (Fig. 3C). Lysophospholipids showed a similar tendency as diacyl phospholipids whereas ether-phospholipids (PL-O), whose biosynthesis takes place in peroxisomes, presented different regulation. In the MCD group, the relative amount of PC-O was significantly increased, while PE-O, PI-O and PS-O were decreased (Supplementary Fig. S3C). The MCD diet also profoundly altered fatty acid chain length and level of desaturation and thus their relative abundance (Supplementary Fig. S4). To evaluate the degree of desaturation, we compared the levels of phospholipids that have between 2–6 double bonds in their two fatty acyl chains (referred to as polyunsaturated fatty acids (PUFA)) to those that have only 1 double bond (referred to as monounsaturated fatty acids (MUFA)). In line with the drastic reduction in the expression of the desaturase enzyme SCD-1 (Fig. 2), we observed a two-fold reduction in PL containing MUFAs (Fig. 5D), mostly due to the depletion in MUFA PC (Fig. 5F), but also in MUFA PI (Fig. 5H) phospholipids. Even though the total content of PL containing 2–6 doubles bonds was not increased (Fig. 5D), livers of MCD-fed mice were enriched in PE, PI and PS containing PUFA PL (Fig. 5G–I). Phospholipids with a combined carbon number of 28–34 in both fatty acyl chains, called long chain (LC) lipids were significantly less abundant in the MCD group compared to the CTRL and ATH groups mostly due to a strong decrease in PC LC lipids (Fig. 5E,F,H). In contrast, PE, PI and PS with 38-44 carbons, called very long chains (VLC) phospholipids were increased (Fig. 5G–I) while PC VLC lipids did not significantly change. Conjointly, this results in a rise of the VLC/LC ratio for PC, PE and PI (Supplementary Fig. S4I–K).

In summary, choline deficiency dramatically modified the relative abundance of the different phospholipid classes, strongly affecting the PC/PE ratio. MCD livers became enriched in phospholipids containing ethanolamine, inositol or serine head groups bound to fatty acids with very long and polyunsaturated fatty acyl chains. In contrast, hepatic phospholipids were less impacted by the ATH diet and only PE containing MUFAs were significantly altered compared to the CTRL group.

## Discussion

In the present study, we combine histological, transcriptional and lipidomic approaches to dissociate common and specific alterations induced by two classical NASH-inducing models, namely the atherogenic diet and methionine and choline deficient diet. We showed that both ATH and MCD diets increased liver lipid content to a similar extent. However indicators of NASH severity, in particular levels of circulating liver enzymes and fibrosis markers (representing the most important histological predictor of disease-related mortality in humans) differed between the two diets. Our transcriptional investigations indicate that the balance between extra-cellular matrix (ECM) production and degradation is dysregulated in both NASH models, but genes involved in ECM remodeling (TIMPSs and MMPs), ECM production (Collagens) as well as in HSC activation (Osteopontin) are more dysregulated in MCD-fed mice than in ATH-fed mice. Interestingly, Bmp6 and Bmp7 that have anti-fibrotic functions are differently regulated in our two NASH models. On one hand, *Bmp6* that has been shown to counteract HSC activation^32^ is significantly downregulated in ATH-fed mice. On the other hand, *Bmp7* is drastically and specifically abrogated in MCD-fed mice. Bmp7 may balance TGF-β production/signaling and have an antifibrotic action^33–35^. Indeed, its overexpression in primary HSC reduces the expression of fibrogenic markers^36^, while adeno-associated overexpression suppresses chemically-induced hepatic fibrosis^36,37^. Thus, the decrease in Bmp6 and Bmp7 expression could contribute to the pathological fibrosis respectively in ATH and MCD models.

To highlight the similarities and specificities of both NASH models in terms of lipid composition, we performed an extensive lipidomic analysis of liver tissue from ATH- and MCD-fed mice. Hierarchical clustering and principal component analysis pointed to elevated sphingolipid (SL) levels as a common feature of both dietary NASH models. Hepatic SL are known to contribute to inflammation, insulin resistance and oxidative stress^38,39^, but can have rather opposite biological functions given the biochemical nature of their head group or their fatty acyl chain length^40–42^. The upregulation of serine palmitoyltransferase *Sptlc2* and ceramide synthases *CerS4-6*, as well as the enrichment in dihydroceramides suggests an increase in *de novo* SL synthesis in both NASH models. Ceramide synthases (CerS) convert sphinganine into dihydroceramides with each CerS having a high specificity towards a selected acyl CoA chain length^30^. In particular, CerS6 shows high substrate preference for palmitoyl(C16:0)-CoA^43^, which generates the toxic C16 ceramide^44–49^ that was 2x and 2.6x more abundant in ATH and MCD groups, respectively, than in the CTRL group (Fig. 4C). This result is in line with the upregulation of CerS6 on a transcriptional level. In contrast, expression of CerS2, which has a preference for very long chain C22-C24 acyl CoAs was not significantly altered, resulting in only a modest relative increase in hepatic levels of the corresponding ceramide species (C22-C24). These observations corroborate previous studies in diet induced obesity (DIO) models, db/db mice and other genetically modified rodents^44–49^. Note that the overexpression of sphingomyelin phosphodiesterase 1 (Smpd1) suggests that ceramides are also enriched by sphingomyelin (SM) hydrolysis in the MCD model. Curiously, choline deficiency does not decrease the amount of SM in the liver of MCD-fed mice as expected and observed *in vitro*^50^ and *in vivo*^21^ and are even increased by 1.9x in the MCD group compared to the CTRL group. This suggests that SM synthesis is preserved thanks to restricted pool of choline originating from endogenous stores. Quantification of the Smpd1 paralogues as well as the two sphingomyelin synthases may decipher this conundrum. In humans, NAFLD/NASH patients present increased plasmatic levels of ceramides^51,52^ and sphingomyelins^53^ and SL are key biomarkers for the progression of NAFLD^54^. One third of the lipid species that present significant differences across the NAFLD states in the plasma and the liver were SL. Using linear discriminant analysis, Gorden, *et al*.^54^ showed that SL is the best lipid class to differentiate NAFLD states and that dihydroceramides are the strongest biomarkers to distinguish steatosis from steatohepatitis. Consistently, Luukonnen and colleagues^55^ showed that almost all ceramide and dihydroceramide species were increased in livers of patients with metabolic NAFLD (high HOMA-IR) compared to controls.

In contrast to SL, phospholipids were differently impacted by the ATH and MCD diets. Similarly to other studies^56,57^, our lipidomic analysis confirmed that the ratio between the two most abundant lipid classes (PC/PE) is strongly reduced by choline deficiency. However, the PC/PE ratio was only slightly impacted by the ATH diet. Li and colleagues^57^ showed that a reduction of the PC/PE ratio coincides with circulating ALT levels and is involved in the progression from steatosis to steatohepatitis. Moreover, PC are crucial for the maintenance of cell membrane integrity^57^ and are decreased in NAFLD and NASH human livers^58^. Lowering of the PC/PE ratio can be also achieved by reducing PC biosynthesis through a deficiency in phosphatidylethanolamine N-methyltransferase that leads to reduced VLDL secretion^59^. In contrast, PC levels can be stabilized by a partial or total disruption of the Mdr2/Abcb4 transporter^57,60^, which transfers PL from hepatocytes to the bile. This potentially protective mechanism is not activated in our context since MCD-fed mice displayed unchanged Mdr2/Abcb4 gene expression compared to control mice. Moreover, the MCD diet affects the degree of fatty acid desaturation. We observed a severe downregulation of the stearoyl-CoA desaturase (*Scd1*) concomitant with a reduction of lipids which contain one double bond in their two fatty acyl chains combined (called MUFA) resulting in a decreased monounsaturated to saturated (MUFA/SFA) ratio (Supplementary Fig. S4). These results are consistent to what has been shown by Rizki, *et al*.^20^, while Larter, *et al*.^17^ presented an increase of the MUFA/SFA ratio in mice fed an MCD diet in combination with lard or olive oil. This difference could be due to the different lipid composition of the diet (corn oil: our study and^20^ *vs* lard or olive oil), or to the sex of the mice (male mice: our study and^20^ *vs* female mice^17^). Finally, our study of the liver PL content revealed an increase in lipids with 2–6 double bonds (PUFA PE, PI and PS), which may be attributed to the fat composition of the MCD diet or to an increase in Δ6 and Δ5 desaturases as reported by Larter, *et al*.^17^. In human NASH, lipidomic analysis of liver biopsies also highlighted dysregulations in the synthesis pathway of fatty acids^61^. Especially, a respectively decreased and increased enzymatic activity of the elongase ELOVL6 and the desaturase FADS1 led to accumulation of long-chain fatty acids and diminution of very long chain fatty acids generating a highly toxic mixture for hepatocytes^61^.

Belong mass spectrometry analysis, our histological observations corroborate what has been described in the literature. In fact, macrosteatosis, which correlates mainly with triglycerides accumulation^17,19–21^, is more frequent in MCD-fed mice, while microsteatosis, representative of cholesterylester accumulation^18,22,62^, is more pronounced in ATH-fed mice. Cholesterol and derivatives such as bile acid (BA) and oxysterol are toxic for the liver when present in excess. In ATH-fed mice, cholesterogenic genes were dramatically downregulated, while the apical export of cholesterol was upregulated, reflecting the expected accumulation of cholesterol in the liver. Cholesterol accumulation can be alleviated by the production of BA and their export into the bile. Albeit, the classic (neutral) pathway of BA synthesis was almost abrogated in the ATH group due to the strong feedback inhibition of dietary cholic acid on 12*-*alpha-hydroxylase (*Cyp8b1*) expression^63^. In contrast, the alternative (acidic) pathway of bile acid synthesis was upregulated in the ATH group, in line with the expected accumulation of oxysterols. These observations mimic situations in mice and humans, in which the BA synthesis pathways are uncoupled^64,65^. In contrast, the livers of MCD-fed mice displayed a concomitant drastic shutdown of both the classic and alternative BA synthesis pathways. In line with Tanaka, *et al*.^66^, we observed a decreased expression in BA basolateral importers and an increased expression of BA basolateral exporters. These coordinated repressions of hepatic BA synthesis and reuptake and stimulation of BA efflux could be initiated by inflammatory signals to limit BA overload^67,68^.

**Figure 6:**
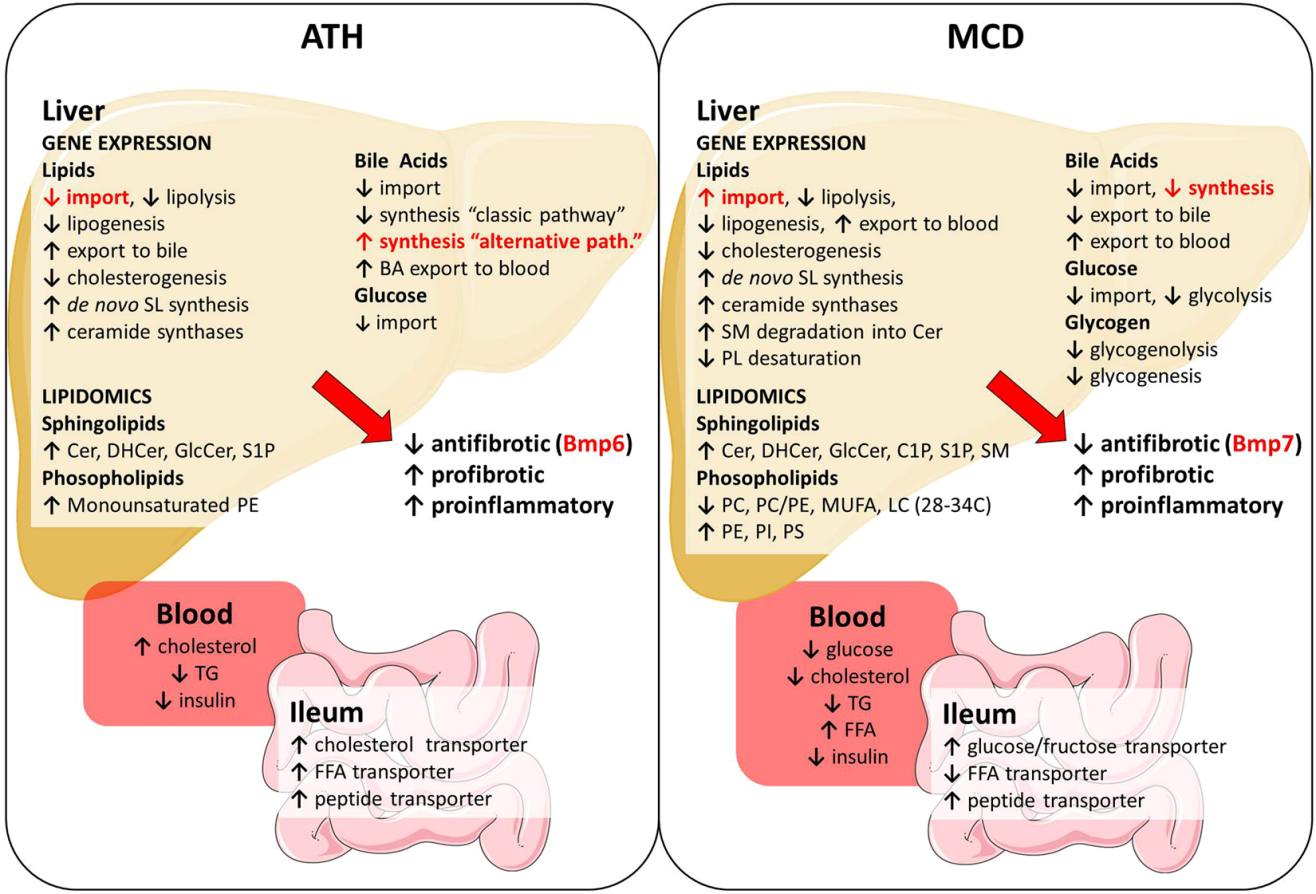
Transcriptional and lipidomic approaches highlights common and specific alterations in ATH- and MCD-fed mice. Illustration of the main alterations of gene expression (liver and ileum), liver lipid content and blood parameters induced by the ATH diet (left panel) and the MCD diet (right panel). In red: alterations that are divergent in the two dietary-induced NASH models. Unmodified drawings were downloaded from the free medical image database Servier Medical Art. Servier Medical Art by Servier is licensed under a Creative Commons Attribution 3.0 Unported License.

In conclusion, ATH or MCD diets represent two complementary dietary models that rapidly recapitulate the liver manifestations of the human NASH disease. Shared and model-specific pathophysiological alterations leading to NASH are summarized in Fig. 6. In accordance to the multiple hit hypothesis, livers undergo numerous insults, such as lipotoxicity, ROS and BA overload leading to hepatic stellate cells (HSC) activation, sustained inflammation and unbalanced homeostasis of the extra-cellular matrix towards collagen deposition. Despite similar degrees of lipid accumulation, ATH and MCD models displayed different degrees of NASH severity questioning about lipid species involved in lipotoxicity. Our lipidomic and gene expression analyses highlight a global dysregulation of SL metabolism upon both NASH models. In line with the observation that SL and in particular dihydroceramides are biomarkers of NAFLD disease state^54^, our study pinpoints to *de novo* ceramide synthesis as a potential target to prevent the progression from simple steatosis to steatohepatitis. Therefore, inhibition of SL biosynthesis by myriocin treatment (a serine-palmitoyl transferase inhibitor with known anti-atherogenic^69–71^ and hepato-protective properties^44–46,72,73^) in ATH and MCD models would be of particular interest to mechanistically delineate the implication of SL in NASH emergence and progression.

## Methods

### Animals

All experiments on animals were approved by the Geneva Canton ethical regulation authority (authorization: GE/202/17) and were conducted according to the Swiss law. Ten-week-old C57BL/6J male mice from Charles River Laboratories were housed under normal conditions and had *ad libitum* access to regular chow diet (CTRL: RM3, Special Diet Services (SDS), 11.5% fat, 61.57% carbohydrate, 26.93% protein) and water for one week of acclimation.

### Diets

Methionine choline deficient (MCD: 21% fat, 63% carbohydrate, 16% protein) and atherogenic (ATH: 60% fat, 20% carbohydrate, 20% protein, 1.25% cholesterol and 0.5% cholic acid) diets were purchased from Research Diets Inc. (A02082002BR, D17052505). Mice were randomly distributed into three different groups (CTRL, MCD, ATH). Mice in the CTRL and ATH groups were fed with their respective diets for 12 weeks. As the MCD diet rapidly induces the liver manifestation of NASH as well as an important weight loss, mice in the MCD group were fed a CTRL diet for 5 weeks followed by 7 weeks of MCD diet.

### Tissue collection

Mice were fasted 4 hours (9a.m.–1p.m.), briefly anesthetized with isoflurane and immediately sacrificed. Truncal blood was collected, and organs were weighed prior to fixation or cryopreservation in liquid nitrogen.

### Histology

Liver, epididymal white adipose tissue (eWAT), inguinal white adipose tissue (iWAT), brown adipose tissue (BAT) and ileum were fixed overnight in 10% formalin and processed for histology. 5-μm sections were rehydrated and either stained with H&E for gross morphology, with Sirius Red/Fast Green for fibrosis quantification or with Periodic Acid Schiff (PAS) for glycogen assessments. To quantify lipid deposition, unfixed pieces of liver were embedded in optimal cutting temperature (OCT) compound and flash frozen. 5-μm sections were rapidly fixed in 10% formalin and stained with hematoxylin and Oil Red O solution.

### Quantification and pathological score

Pictures were acquired using an Olympus VS120 microscope. Fibrosis, lipid and glycogen quantifications were performed with the area measurement tool of the ImageJ software^74^. Ileal villus and crypt sizes were measured on H&E stained sections with the ImageJ software. The SAF score (Steatosis, Activity (ballooning and lobular inflammation) and Fibrosis) was evaluated by a pathologist (CdV) blinded to the diets according to Bedossa, *et al*.^75^.

### RT-qPCR

Total RNA was isolated from liver and ileum tissues using TRI Reagent Solution (ThermoFisher AM9738). 500 ng of total RNA were reverse transcribed (PrimeScript RT reagent kit, Takara) and mRNA levels were assessed by qPCR using a Light-Cycler 480 (Roche Diagnostics). Normalization was done using the ribosomal *RPS29* gene. The list of primers can be found in Supplementary Table S1.

### Blood parameters

Blood samples were collected in EDTA-coated tubes and plasma was stored at −80 °C. Plasma levels of glucose, AST, ALT, total cholesterol, free fatty acids and triglycerides were assessed using a Cobas c 111 analyzer and appropriate reagents (Roche Diagnostics). Insulin was measured using an ultrasensitive mouse insulin ELISA kit (Mercodia).

### Lipidomic analysis

Lipid extraction and lipidomic analysis were performed as described in Loizides-Mangold, *et al*.^76^. Lipid extracts were prepared using the MTBE protocol^77^. In short, 15–20 mg of pulverized liver tissue was resuspended in 100 µl H_2_O. 360 μl methanol and a mix of internal standards were added (400 pmol PC 12:0/12:0, 1000 pmol PE 17:0/14:1, 1000 pmol PI 17:0/14:1, 3300 pmol PS 17:0/14:1, 2500 pmol SM d18:1/12:0, 500 pmol Cer d18:1/17:0 and 100 pmol GlcCer d18:1/8:0) together with 1.2 ml of MTBE. Phase separation was induced by addition of 200 µl MS-grade water and the organic phase was dried in a vacuum concentrator (CentriVap, Labconco). Lipids were dissolved in chloroform/methanol and divided into three aliquots. One aliquot was treated by alkaline hydrolysis to enrich for sphingolipids according to the method by Clarke and Dawson^78^ and the other two aliquots were used for glycerophospholipids and phosphorus assay, respectively. Mass spectrometry analysis for the identification and quantification of phospho- and sphingolipid species was performed on a TSQ Vantage Triple Stage Quadrupole Mass Spectrometer (Thermo Fisher Scientific) equipped with a robotic nanoflow ion source (Nanomate HD, Advion Biosciences), using multiple reaction monitoring (MRM). Each individual ion dissociation pathway was optimized with regard to collision energy. Dried lipid extracts were resuspended in 250 µl MS grade chloroform/methanol (1:1) and further diluted in either chloroform/methanol (1:2) plus 5 mM ammonium acetate (negative ion mode) or in chloroform/methanol/H_2_O (2:7:1) plus 5 mM ammonium acetate (positive ion mode). Lipid concentrations were calculated relative to the relevant internal standards and normalized to the phosphate content of each lipid extract to account for variability in the amount of starting material and to correct for sample loss during the extraction procedure.

### Statistical analyses

GraphPad Prism version 7.02 software was used for statistical analyses of the data using one-way ANOVA, p < 0.05 was considered statistically significant. Graphs represent mean + standard error of the mean (SEM), if not stated differently. Clustered heat map was generated in R using the heatmap.2 function of the “gplots” package. Principal component analysis was performed using the PAST program using default parameters^79^.

## Supplementary information

Supplementary table S1, Supplementary figure S1, Supplementary figure S2, Supplementary figure S3, Supplementary figure S4
