# Supplementary material for: Multi-technique comparison of atherogenic and MCD NASH models highlights changes in sphingolipid metabolism
Source: Sci Rep. 2019 Nov 14;9:16810. doi: 10.1038/s41598-019-53346-4 (PMC6856196; doi:10.1038/s41598-019-53346-4)
Supplement: Supplementary file 1 — Supplementary table S1, Supplementary figure S1, Supplementary figure S2, Supplementary figure S3, Supplementary figure S4 [file 41598_2019_53346_MOESM1_ESM.docx]

**Title**:

Multi-technique comparison of atherogenic and MCD NASH models highlights changes in sphingolipid metabolism.

**Authors**:

Sophie A. Montandon^1^, Emmanuel Somm^1^, Ursula Loizides-Mangold^1,2,3,4^, Claudio de Vito^5^, Charna Dibner^1,2,3,4^ and François R. Jornayvaz^1*^

**Supplementary table S1. List of forward and reverse primers used for RT-qPCR in the liver and ileum.**

| **Gene name** | **Forward** | **Reverse** |
| --- | --- | --- |
| Abcb11 (BSEP) | CTGCCAAGGATGCTAATGCA | CGATGGCTACCCTTTGCTTCT |
| Abcb4 (MDR2/3) | GCAGCGAGAAACGGAACAG | GGTTGCTGATGCTGCCTAGTT |
| Abcc4 (MRP4) | GCAGCGAGAAACGGAACAG | GGTTGCTGATGCTGCCTAGTT |
| Abcg5 | TGGATCCAACACCTCTATGCTAAA | GGCAGGTTTTCTCGATGAACTG |
| Abcg8 | CGTCGTCAGATTTCCAATGA | GGCTTCCGACCCATGAATG |
| Acta2 (αSMA) | TCCTCCCTGGAGAAGAGCTAC | TATAGGTGGTTTCGTGGATGC |
| Adgre1 (F4/80) | CTTTGGCTATGGGCTTCCAGTC | GCAAGGAGGACAGAGTTTATCGTG |
| Bmp6 | CCCACTCAACGCACACATG | GGATTCATAAGGTGGACCAAGGT |
| Bmp7 | CAAGACGCCAAAGAACCAAGAG | GGTCTCGGAAGCTGACGTACAG |
| Cat | CAGATGAAGCAGTGGAAGGAG | CGTCAGTGAAAACCACATCCT |
| Ccl2 (MCP-1) | TTAAAAACCTGGATCGGAACCAA | GCATTAGCTTCAGATTTACGGGT |
| Cd36 | AGATGACGTGGCAAAGAACAG | CCTTGGCTAGATAACGAACTCTG |
| CerS2 | TCTACTGGTCCCTGCTCTTCA | CCAGGAGAAGCAGAGGAGAAT |
| CerS4 | CAGTTTCCTGCATAAGGGTCA | CAGCTGTGGACCTTCAGAAAC |
| CerS5 | CATGCCATCTGGTCCTACCTA | CTGCCAGAGAGGTTGTTTTTG |
| CerS6 | AAGGGTTGAACTGCTTCTGGT | AGTGTGGTTTCTTCCCTGGAG |
| Col1a1 | ACGTCCTGGTGAAGTTGGTC | CAGGGAAGCCTCTTTCTCCT |
| Col3a1 | CCACTCTTATTTTGGCACAGC | ATGTCATCGCAAAGGACAGAT |
| Col4a1 | ACTGTGGATCGGCTATTCCTT | GGCGCTTCTAAACTCTTCCAG |
| Cpt1a | AACCCAGTGCCTTAACGATG | GAACTGGTGGCCAATGAGAT |
| Cyp7a1 | GCCTCTGAAGAAGTGAATGG | CTAGTACTGGCAGGTTGTTTAG |
| Cyp7b1 | CTGCAGTCAACAGGTCAAA | GCCTCAGAACCTCAAGAATAG |
| Cyp8b1 | CTGGCTTCCTGAGCTTATTC | CCCAGTAGGGAGTAGACAAA |
| Ddit3 (CHOP) | AGCGACAGAGCCAGAATAACA | TCAGGTGTGGTGGTGTATGAA |
| Degs1 (DES1) | TCACCTTCAATGTGGGCTATC | CTCACTTGCGATCTTCCTCAC |
| Dgat2 | AGTGGCAATGCTATCATCATCGT | AAGGAATAAGTGGGAACCAGATCA |
| Fabp6 | GCCTTCAGTGGCAAATATGAA | TTCAATCACGTCTCCTGGAAG |
| Fasn (FAS) | CATGACCTCGTGATGAACGTGT | CGGGTGAGGACGTTTACAAAG |
| Gck | ACTTGAGGCAGCTATGTGCAGG | GCTGTCTCCAGAATCTGTGTACTG |
| Gpx1 | GGACTACACCGAGATGAACGA | CAATGTAAAATTGGGCTCGAA |
| Gpx3 | TCTACGAGTATGGAGCCCTCA | AATGGCCCAAGTTCTTCTTGT |
| Gys2 | ACCAAGGCCAAAACGACAG | GGGCTCACATTGTTCTACTTGA |
| Hmgcr | CTTGTGGAATGCCTTGTGATTG | AGCCGAAGCAGCACATGAT |
| Hmgcs1 | GCCGTGAACTGGGTCGAA | GCATATATAGCAATGTCTCCTGCAA |
| Hspa5 (GRP78/Bip) | ACTTGGGGACCACCTATTCCT | ATCGCCAATCAGACGCTCC |
| IL1b | ACTGTGAAATGCCACCTTTTG | TTTGAAGCTGGATGCTCTCAT |
| Itgax (Cd11c) | CACTCAGTGACTGCCCAAAA | CCTCAAGACAGGACATCGCT |
| Ldlr | TCCAATCAATTCAGCTGTGG | GAGCCATCTAGGCAATCTCG |
| Mmp13 | AGGCCTTCAGAAAAGCCTTC | TCCTTGGAGTGATCCAGACC |
| Mmp14 | CCGCCATGCAAAAGTTCTAT | GCCCACCTTAGGGGTGTAAT |
| Mmp2 | GTCGCCCCTAAAACAGACAA | GGTCTCGATGGTGTTCTGGT |
| Mmp3 | ACGATGGACAGAGGATGTCAC | AGATGGAAACGGGACAAGTCT |
| Mmp9 | GCAGAGGCATACTTGTACCGCTAT | CGGCCGTAGAGACTGCTTCT |
| Mttp | ATGATCCTCTTGGCAGTGCTT | TGAGAGGCCAGTTGTGTGAC |
| Nox4 | ACCCAAGTTCCAAGCTCATTT | ATGGTGACAGGTTTGTTGCTC |
| Npc1l1 | GAAGCACAGCTGACAAGTTCC | CTGGATGGTAGTGCGATTGAT |
| Pck1 (Pepck) | CTGCATAACGGTCTGGACTTC | GCCTTCCACGAACTTCCTCAC |
| PDGFc | TGAGAGATTTGGGCTGGAAGA | ACTGGGCTCCTCAACTTCTACAA |
| Pfkl | CCATGTTGTGGGTGTCTGAG | ACAGGCTGAGTCTGGAGCAT |
| Pnpla2 (Atgl) | GTCCTTCACCATCCGCTTGTT | CTCTTGGCCCTCATCACCAG |
| PPARa | CAGGAGAGCAGGGATTTGCA | CCTACGCTCAGCCCTCTTCAT |
| Pygl | GAGAAGCGACGGCAGATCAG | CTTGACCAGAGTGAAGTGCAG |
| RPS29 | GCCAGGGTTCTCGCTCTTG | GGCACATGTTCAGCCCGTAT |
| Scd1 | CCTTCCCCTTCGACTACTCTG | GCCATGCAGTCGATGAAGAA |
| Slc10a1 (NTCP) | ATGACCACCTGCTCCAGCTT | GCCTTTGTAGGGCACCTTGT |
| Slc15a1 | AATGACCTCACAGACCACGAC | TCCTCCTGTACCAAGGGCTAT |
| Slc2a2 (Glut2) | GTCCAGAAAGCCCCAGATACC | GTGACATCCTCAGTTCCTCTTAG |
| Slc2a5 | CTACATGATCGGAGGCAGTGT | ATGGTGGTGAGGAAACAGATG |
| Slc51b (OST-β) | GTATTTTCGTGCAGAAGATGCG | TTTCTGTTTGCCAGGATGCTC |
| Slc5a1 | TTCTGTCCCTGCTCCTCTACA | CAGGATGAAAATGGCCAAGTA |
| Slco1a1 (OATP-1) | CAGTCTTACGAGTGTGCTCCAGAT | ATGAGGAATACTGCCTCTGAAGTG |
| Smad2 | CCGTACCACTACCAGAGAGTTGAG | GGCGGCAGTTCTGTTAGAATCT |
| Smad3 | CCAATGTCAACCGGAATGC | CGCACACCTCTCCCAATGT |
| Smad4 | GGAGCTCATCCTAGCAAGTGTGT | CCGACCAGCCACCTGAAGT |
| Smad5 | TGGATCTAAGCAAAAGGAAGTTTGT | CACTAATACTGGAGGTAAGACTGGACTCT |
| Smad6 | TATTCTCGGCTGTCTCCTCC | AGTGATGAGGGAGTTGGTGG |
| Smpd1 | GGCTCTTGATCAACTCCACAG | GACAATGTCCTGGAGGGATGT |
| Sod1 | GAGACCTGGGCAATGTGACT | GTTTACTGCGCAATCCCAAT |
| Sod2 | CCGAGGAGAAGTACCACGAG | GCTTGATAGCCTCCAGCAAC |
| Spp1 | AAAGCACCCTGTAGAAAACA | CCGTTTTATCCTCTCTACACTC |
| Sptl1c (SPT) | GTCCCTCCAGTCTCCAAGAAC | AGCAGCCCAAGGAAGTTAAAG |
| Srebf1c (Srebp-1) | GGAGCCATGGATTGCACATT | GGCCCGGGAAGTCACTGT |
| Srebf2 (Srebp2) | GGCGCCCCACTCAGAACACC | CAGGAACGGCGGTCACCCAC |
| sXBP1 | CTGAGTCCGCAGCAGGTG | TGAAAAACATGACAGGGTCCA |
| Tgfb1 | AACAATTCCTGGCGTTACCTT | GAATCGAAAGCCCTGTATTCC |
| Tgfb2 | GGATCTTGGATGGAAATGGAT | ATGGTGTTGTACAGGCTGAGG |
| Timp1 | ATTCAAGGCTGTGGGAAATG | CTCAGAGTACGCCAGGGAAC |
| Timp2 | TCCTTGCTACAGGCAGGAGT | CATTCGCTGAAGTCTGTGGA |
| Tnf (TNFα) | CAGCCTCTTCTCATTCCTGCT | TCTGGGCCATAGAACTGATGA |
| Vim (Vimentin) | GGAGGAGATGCTCCAGAGAGA | TTCCTGCAAGGATTCCACTTT |
| Vldlr | CTCCCAGTTTCAGTGCACAA | ATCAGAACCGTCTTCGCAAT |

**Supplementary figure S1**. **Histological sections of adipose tissues stained with hematoxylin and eosin (H&E).**


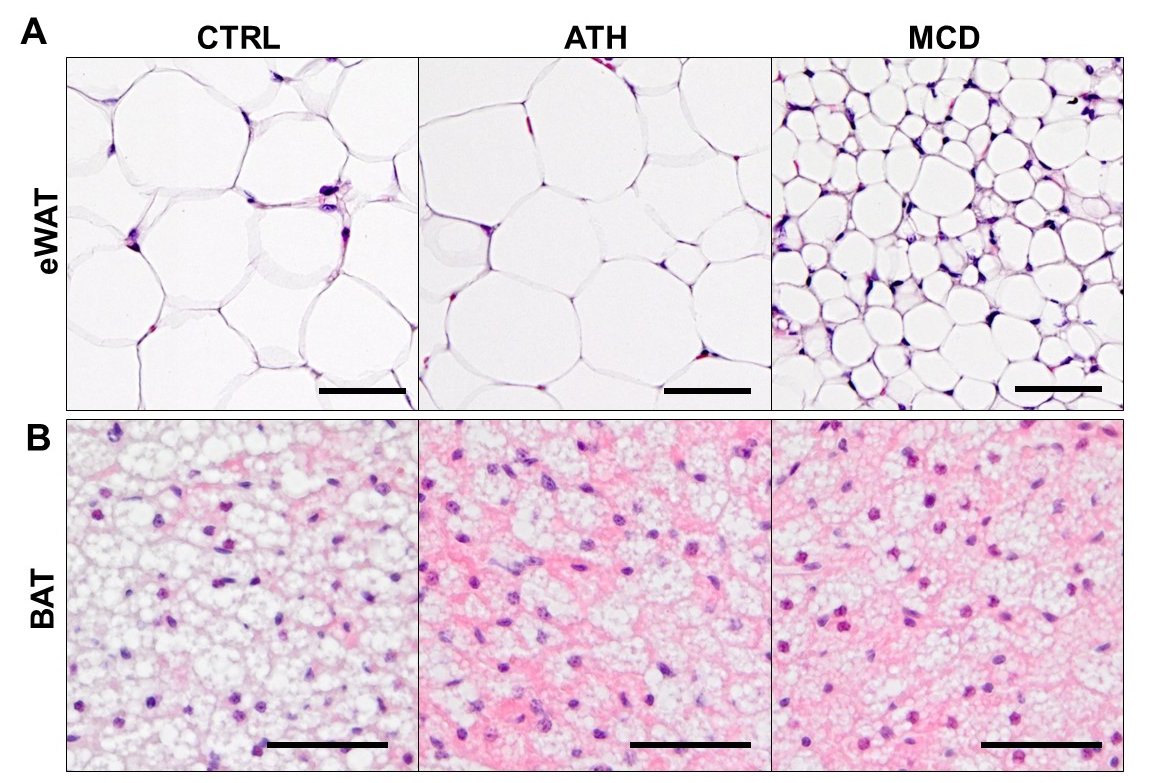


Epididymal white adipose tissue (A) and brown adipose tissue (B) sections. Scale bars 50 μm.

**Supplementary figure S2. ATH and MCD diets alter nutrient absorption.**


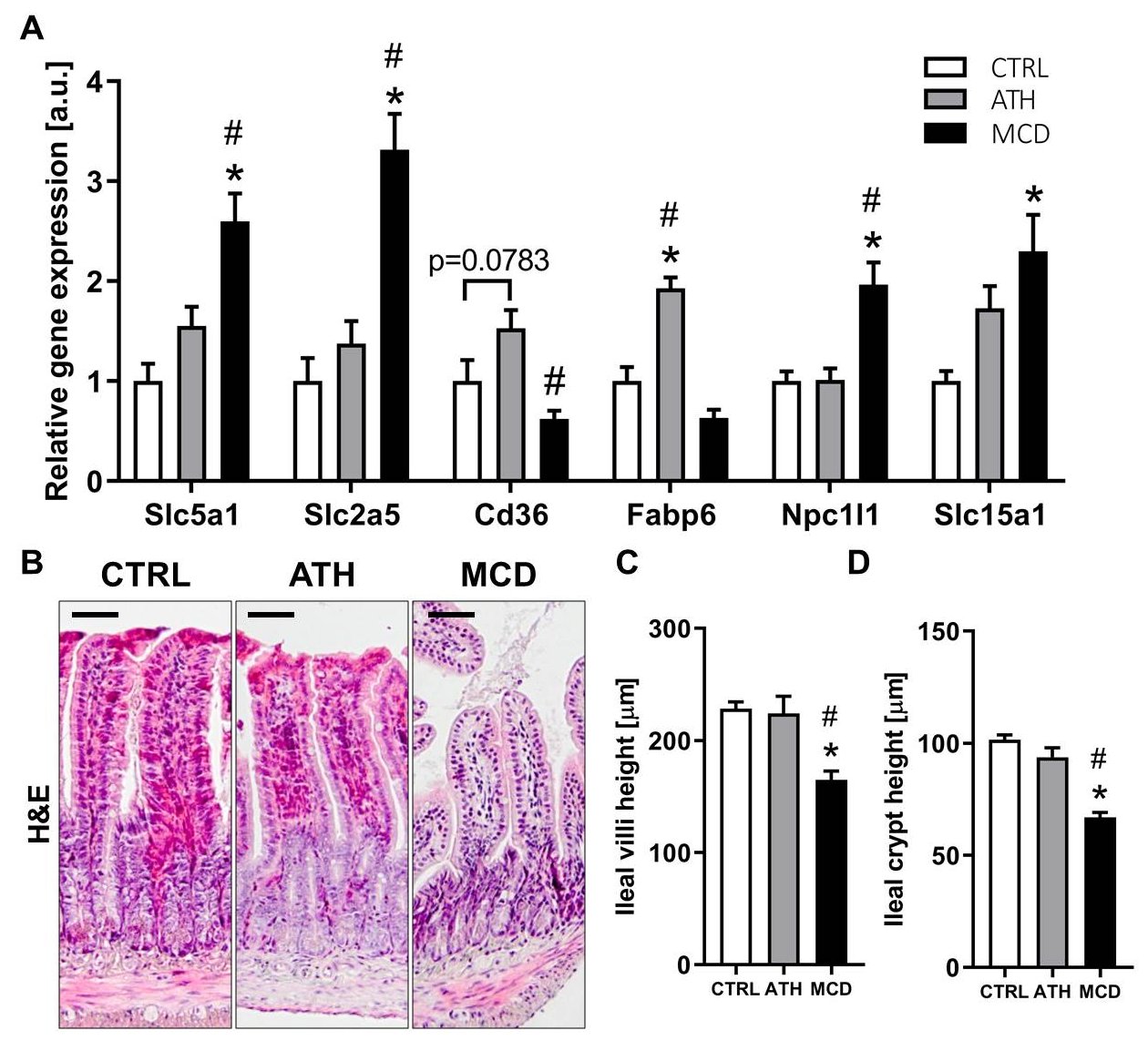


RT-qPCR of nutrient transporters (A). Histological sections of ileum stained with H&E (B). Measurements of ileal villi height (C) and crypts height (D). Scale bar 50 µm. One-way ANOVA: * p value ATH/MCD *vs* CTRL < 0.05, # p value MCD *vs* ATH < 0.05.

**Supplementary figure S3. Relative abundance of diacyl-, lyso- and ether-containing phospholipids.**


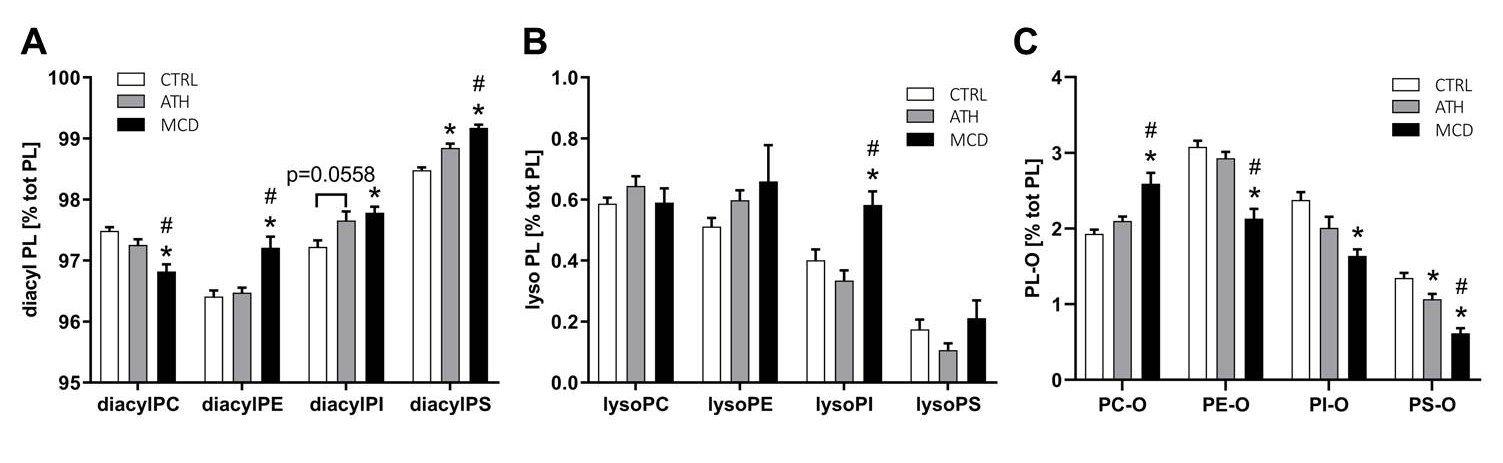
Relative concentration of diacyl- (A), lyso- (B) and ether-containing phospholipids (C). PL: phospholipids, PC: phosphatidylcholine, PE: phosphatidylethanolamine, PI: phosphatidylinositol, PS: phosphatidylserine, PL-O: ether-containing phospholipids. One-way ANOVA: * p value ATH/MCD *vs* CTRL < 0.05, # p value MCD *vs* ATH < 0.05.

**Supplementary figure S4. Ratios of phospholipid concentration according to their fatty acid chain length or level of unsaturation.**


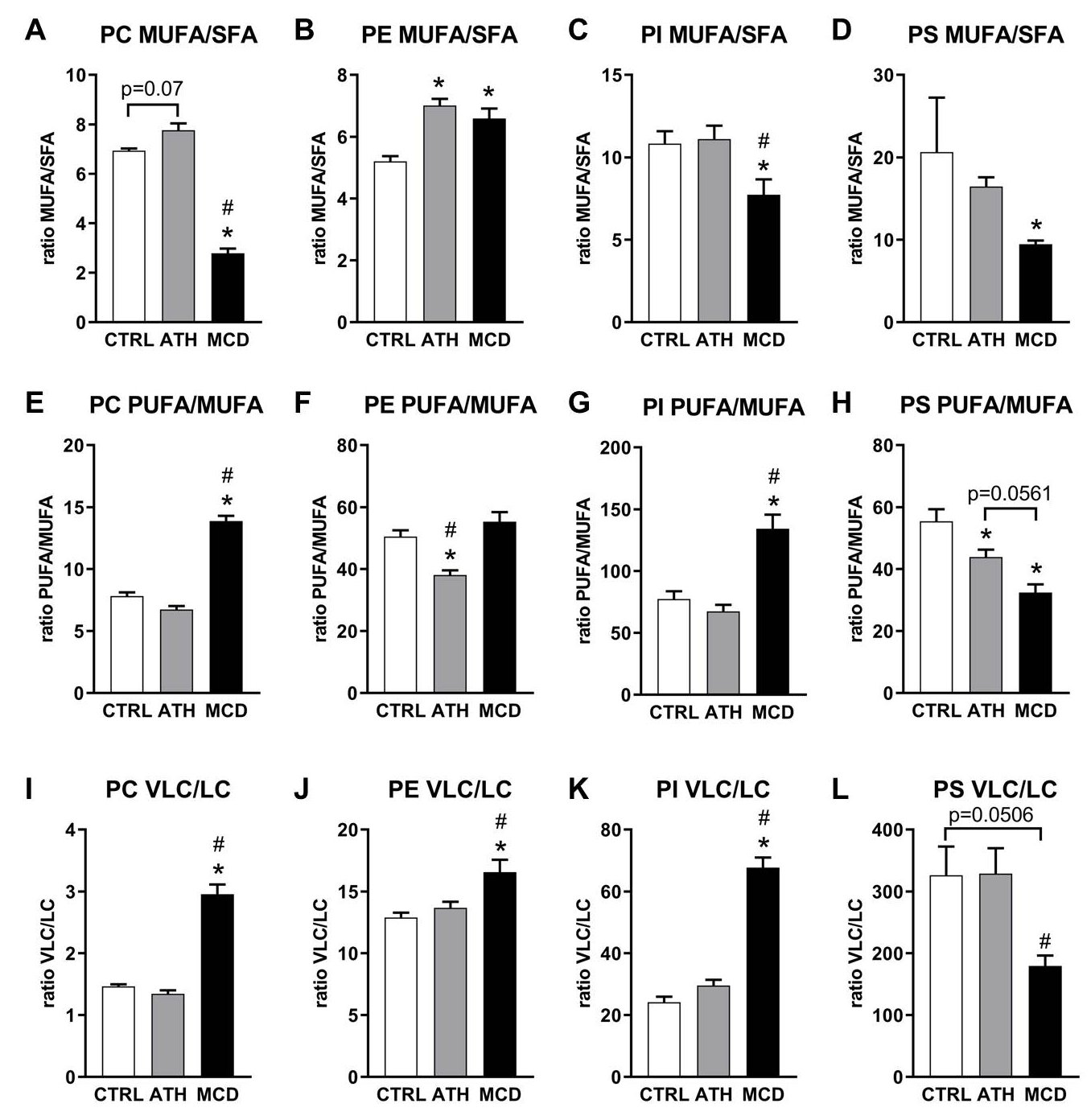


Ratios of monounsaturated fatty acids (MUFA) to saturated fatty acids (SFA) of phospholipids with choline (A), ethanolamine (B), inositol (C) or serine (D) head group. Ratios of polyunsaturated fatty acids (PUFA) to MUFA, with choline (E), ethanolamine (F), inositol (G) or serine (H) head group. Ratios of very long fatty acyl chains (VLC) to long fatty acyl chains (LC) of phospholipids with choline (I), ethanolamine (J), inositol (K) or serine (L) head group. One-way ANOVA: * p value ATH/MCD *vs* CTRL < 0.05, # p value MCD *vs* ATH < 0.05.
